# Incorporation of liver chemistry score in predicting survival of liver‐involved advanced gastric cancer patients who received palliative chemotherapy

**DOI:** 10.1002/cam4.5179

**Published:** 2022-09-04

**Authors:** Ying Feng, Cheng Zhang, Zhijun Wu, Hui Xu, Xiaopeng Zhang, Chong Feng, Jingyi Shao, Minmin Xie, Yahui Yang, Yi Zhang, Tai Ma

**Affiliations:** ^1^ Department of Oncology The First Affiliated Hospital of Anhui Medical University Hefei People's Republic of China; ^2^ Anhui Provincial Cancer Institute/Anhui Provincial Office for Cancer Prevention and Control Hefei People's Republic of China; ^3^ Department of Oncology Ma'anshan Municipal People's Hospital Ma'anshan People's Republic of China; ^4^ Department of Noncommunicable Diseases and Health Education Hefei Center for Disease Control and Prevention Hefei People's Republic of China

**Keywords:** gastric cancer, liver function tests, liver metastasis, survival analysis

## Abstract

**Background:**

Gastric cancer liver metastasis (GCLM) patients usually accompany by abnormal serum liver function tests (LFTs) more or less; however, the prognostic value of LFTs is not fully understood. This study aimed to develop a liver chemistry score (LCS) based on LFTs and incorporate it into prognosis determination for GCLM patients who received palliative chemotherapy.

**Methods:**

Data were derived from hospitalized GCLM patients in two general hospitals in China. LCS was generated based on the results of LFTs by LASSO regression. Cutoff value of the score was determined by restricted cubic spline. The score was then incorporated into Cox regression analysis to construct a predictive nomogram; the model was then evaluated internally and externally by AUC of time‐dependent receiver operating characteristic curves (ROC) and calibration curves.

**Results:**

Three hundred and thirty‐six and 72 patients were included in development and validation cohort, respectively. LASSO regression analysis in development cohort finally reached a two‐parametric LCS calculated on AST and ALP levels as 0.03343515 × ln (AST, U/L) + 0.02687997 × ln (ALP, U/L), and 0.232 was set as optimal cutoff value. Patients in low (LCS < 0.232) or high (LCS ≥ 0.232) score group experienced different survival times; median OS was 13.54 (95% CI: 11.1–15.6) months in the low LCS group and 7.3 (6.6–9.3) months in the high LCS group (*p* < 0.001). A nomogram including LCS and other clinical parameters was constructed and showed superior performance than model not including LCS. AUC of 6‐month ROC improved from 0.647 (95% CI: 0.584–0.711) to 0.699 (0.638–0.759) in internal validation, and 0.837 (0.734–0.940) to 0.875 (0.784–0.966) in external validation.

**Conclusions:**

Liver chemistry score is useful in determining the prognosis of gastric cancer patients with liver metastasis and may be helpful to clinicians in decision‐making.

## Graphical abstract

In this study, serum parameters of liver function tests in patient with gastric cancer liver metastasis (GCLM) were analyzed by lasso regression; a liver chemistry score (LCS) was generated as 0.03343515 × ln (AST, U/L) + 0.02687997 × ln (ALP, U/L). Patients in low (LCS < 0.232) or high (LCS = 0.232) score group experienced different survival times. The score was also demonstrated as an independent prognostic factor for GCLM patients and incorporated into predictive nomogram model.

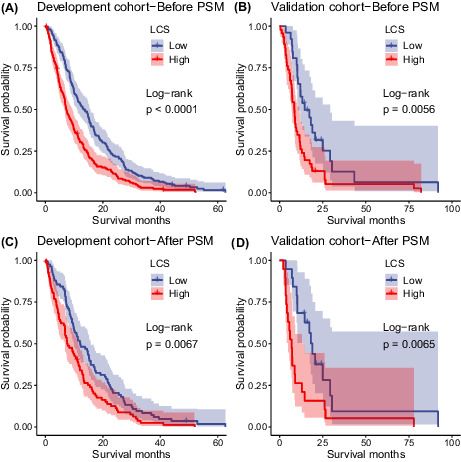

## 1 INTRODUCTION

It is well known that the prognosis of metastatic gastric cancer patients is poor, with a 5‐year survival rate of only 5.3% in the United States and less than 10% in China.^1^, ^2^ The median survival time ranged from 8 to 14 months.^3^, ^4^, ^5^, ^6^ Liver is the most common site of hematogenous metastasis for gastric cancer, which accounts for more than 40% of synchronous distant metastasis.^7^ Moreover, for those without metastasis at initial diagnosis, even after receiving radical gastrectomy, a considerable number of them would experience recurrent events and develop as metachronous liver metastatic disease.^8^ The Surveillance, Epidemiology, and End Results (SEER) cancer database showed that the prognosis of liver‐involved subgroup was likely to be more unfavorable, with median survival time of only 4 months and less than 5% in 5‐year survival.^7^ However, the prognostic factors for this subgroup are still not fully understood.

Liver is the largest metabolic organ, which is responsible for macromolecule synthesis, metabolite biotransformation, and bile secretion. A panel of serum chemistry test, namely liver function tests (LFTs), has been clinically employed to reflect organ function. Elevation of liver enzymes was seen in cancer patients with or without liver metastasis.^9^ Although these serum biochemical parameters are of less diagnostic value in hepatic metastatic cancer,^10^, ^11^ some of them have been demonstrated to be associated with outcomes of gastric cancer and were incorporated into prognostic prediction model, such as alkaline phosphatase,^12^, ^13^, ^14^, ^15^, ^16^, ^17^, ^18^ bilirubin,^14^, ^16^, ^19^ and albumin.^14^, ^15^, ^16^, ^17^ However, the selection of parameters into prognostic model was so far arbitrary; different models included different serum parameters due to reasons such as study objective and data availability. Correlation and collinearity of the laboratory values were seldom taken into consideration. In addition, these models were universal for all advanced patients; more precise determination of prognosis for liver metastatic subgroup is needed.

We hereby hypothesize that the survival outcome of liver metastatic gastric cancer could be determined by profiles of serum liver chemical indicators at liver metastasis. By using Lasso‐penalized analysis of a cluster of 12 common indicators of LFTs, a prognostic liver chemistry scoring system was developed in a retrospective gastric cancer cohort and was then introduced into prognostic nomogram with other clinical and laboratory parameters.

## 2 MATERIALS AND METHODS

### 2.1 Study design and participant enrollment

This two‐center retrospective study consisted of a development cohort and a validation cohort. The development cohort included consecutive patients who were diagnosed as advanced gastric cancer with liver metastasis (GCLM) and received palliative chemotherapy in the First Affiliated Hospital of Anhui Medical University (China) from September 2010 to December 2020; the validation cohort included eligible patients in Ma'anshan Municipal People's Hospital from August 2009 to December 2020. The study protocol conforms to the ethical guidelines of the 1975 Declaration of Helsinki (6th revision, 2008) and was approved by the Ethics Committee of the First Affiliated Hospital of Anhui Medical University (reference number: Quick‐PJ 2021‐05‐19). Due to retrospective design, informed consent was waived.

**FIGURE 1:**
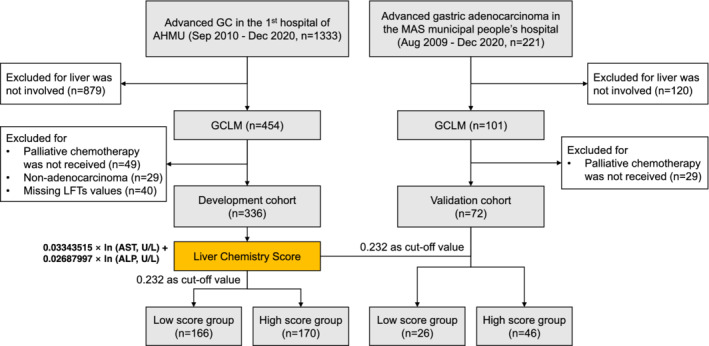
Flow diagram of participant's enrollment. GCLM, gastric cancer liver metastasis; AHMU, Anhui Medical University (China); MAS, Ma'anshan (China); ln, natural logarithm; AST, aspartate aminotransferase; ALP, alkaline phosphatase

The inclusion criteria were as follows: (1) pathologically diagnosed gastric adenocarcinoma; (2) radiologically or pathologically confirmed liver metastasis; (3) clinical information and results of LFTs at the diagnosis of liver metastasis were available; (4) palliative chemotherapy was received. Patients with multiple primary cancers, or incomplete clinical information, or missing value(s) in LFTs were excluded. The flowchart of patients' selection was shown in Figure 1.

### 2.2 Endpoint and patient follow‐up

The endpoint event was death due to any reason. Outcomes of patients were matched in the population death register information system of Anhui province (China), and the death date was recorded. For those unmatched individuals, survival status was obtained by regular telephonic contact to the patients or their relatives. Overall survival (OS) was defined as interval months between the onset of liver metastasis and the death date or the last follow‐up date.

### 2.3 Data sources and variables

Hospital‐stored medical files were accessed to obtain essential clinical and laboratory data. The following clinical variables were documented: (1) patient‐related variables, such as birth date, sex, history of alcohol drinking and cytotoxic drug exposure before liver metastasis, comorbidities of chronic liver diseases including hepatitis virus B or C infection, liver cirrhosis, fatty liver disease, body mass index (BMI), and Eastern Cooperative Oncology Group (ECOG) performance status score at liver metastasis; (2) tumor‐related parameters, including tumor grade, date of liver metastasis, number of liver metastases, carcinoembryonic antigen (CEA) and carbohydrate antigen 19‐9 (CA19‐9) level at liver metastasis, existence of extrahepatic metastasis or not, local treatment aimed to hepatic lesions such as chemotherapy, radiotherapy, surgical resection, and hepatic vascular intervention therapy. The serum LFTs consisted of 12 markers: total protein, albumin, globulin, total bilirubin, direct bilirubin, indirect bilirubin, alanine transaminase, aspartate aminotransferase (AST), alkaline phosphatase (ALP), gamma‐glutamyl transpeptidase, lactate dehydrogenase, and total bile acid. Only those tested within 4 weeks to radiological confirmation of liver metastasis but before commencing of new round of antitumor therapy were eligible in this study.

### 2.4 Development of liver chemistry score

Lasso‐penalized Cox regression analysis was employed to reduce dimensionality of LFTs cluster data as to their impact on survival outcomes. All the values of LFTs were ln‐transformed to reduce skewed distribution and to accord with proportional hazard (PH) hypothesis. Tenfold cross‐validations were used to determine the optimal values of *λ*. A liver chemistry score (LCS) based on the selected indicators was the sum of the regression coefficients (*β*) derived from the Lasso Cox regression model multiplied with its ln‐transformed level. The analysis was performed in R software 4.1.1 (The R Foundation for Statistical Computing, Vienna, Austria) with “glmnet” and “caret” packages.^20^ The score was further divided into a binary factor using the cutoff value indicated by restricted cubic spline (RCS) threshold (“rms” package). Kaplan–Meier survival curves were plotted to test the separation of survival between low score group and high score group both in the development and validation cohort. We also used propensity score matching (PSM) to balance clinical features between two groups if differences across the groups were detected. The subjects were matched 1:1 into each group by the nearest neighbor method (“MatchIt” package).^21^

### 2.5 Univariate and multivariate Cox regression

Before performing Cox regression, numerical variables (CEA and CA19‐9) were transformed into categorical variables; RCS was used to determine the cutoff values as to their impact on survival. The univariate Cox regression was used in the primary screening of prognostic factors in the development cohort. Statistically significant variables were included in the multivariate Cox hazard model analysis. In addition, a multivariate Cox regression without LCS was also performed with aims of comparison with above model. Cox regression was performed in “R” statistics using the “cph” function in “rms” package with. The forward stepwise method was used to select prognostic predictive variables, including parameters with *p* values <0.05, and excluding those with *p* values >0.10. The Cox regression results were described as hazard ratios (HRs) and 95% confidence intervals (CIs). All P values were two‐tailed. *p* values less than 0.05 were considered statistically significant.

### 2.6 Construction and validation of the nomogram model

The nomogram score was calculated using the β coefficient of the Cox regression. The nomogram was plotted using the “rms” and “survival packages” in “R” version 4.1.1. The model was validated both internally in the development cohort and externally in the validation cohort. The discrimination ability was evaluated by investigation of area under time‐dependent ROC curve (“timeROC” package).^22^ Calibration curves were plotted to reflect the discrepancy between nomogram‐predicted 6‐month and 12‐month survival probabilities with the observed survival outcomes. For the internal validation of the nomogram model, 1000 bootstraps with sample sizes of 100 were generated from the development cohort. The external validation dataset included patients in the validation cohort, and 1000 bootstrapping (size 20) was performed.

### 2.7 Other statistical considerations

All statistical description and analysis were performed using R software 4.1.1. Continuous variables were described as median with interquartile range and compared using Wilcoxon test between two groups. Categorized variables were described as number (%) and compared using chi‐square test or Fisher exact test. Kaplan–Meier method was used to plot survival curves and log‐rank test was used to detect statistical significance. A *p*‐value less than 0.05 was considered statistical significant.

## 3 RESULTS

### 3.1 Baseline characteristics of the patients

In the development set, a total of 454 GCLM patients were filtered out from 1333 advanced gastric cancer patients. After excluding 118 ineligible individuals, 336 patients were finally included into analysis. The flow diagram of participant enrollment was illustrated in Figure 1. By December 2020, 329 cases were dead, 4 cases were alive, and 3 patients were lost to follow‐up. The validation cohort included 72 patients whose liver metastases were diagnosed between August 2009 and December 2019. Sixty‐seven patients died and five patients were alive by December 2020.

Baseline characteristics of patients in the two cohorts were shown in Table 1. The OS was comparable between the two cohorts (*p*_log‐rank_ = 0.377). The median BMI was lower in development cohort (*p* = 0.002). Local treatment for hepatic lesion and history of alcohol drinking were more frequently observed in the validation set (*p* < 0.05).

**TABLE 1:** Clinical characteristics of gastric cancer patients with liver metastasis

Variables	Development cohort (*n* = 336)	Validation cohort (*n* = 72)	*p*‐value
Age at liver metastasis (years)	65.0 (58.0, 72.0)	66.0 (61.8, 71.0)	0.202
BMI at liver metastasis (kg/m^2^)	20.56 (18.37, 22.66)	21.66 (19.96, 23.53)	0.002
Median OS (months)	9.86 (8.89, 11.30)	10.22 (8.60, 12.90)	0.377
Sex			
Female	78 (23.21)	15 (20.83)	0.778
Male	258 (76.79)	57 (79.17)	
Grade			
G1‐2	81 (24.11)	22 (30.56)	0.029
G3‐4	188 (55.95)	45 (62.50)	
Unknown	67 (19.94)	5 (6.94)	
Previous gastrectomy			
No	172 (51.19)	43 (59.72)	0.236
Yes	164 (48.81)	29 (40.28)	
ECOG score at liver metastasis			
0~1	268 (79.76)	55 (76.39)	0.631
2~	68 (20.24)	17 (23.61)	
Extrahepatic metastasis			
Absent	101 (30.06)	30 (41.67)	0.076
Present	235 (69.94)	42 (58.33)	
Local treatment for hepatic lesion			
No	301 (89.58)	45 (62.50)	<0.001
Yes	35 (10.42)	27 (37.50)	
History of alcohol drinking			
No/unknown	281 (83.63)	40 (55.56)	<0.001
Yes	55 (16.37)	32 (44.44)	
Chronic liver disease			
No/unknown	327 (97.32)	69 (95.83)	0.451
Yes	9 (2.68)	3 (4.17)	
Previous cytotoxic drugs exposure			
No	246 (73.21)	47 (65.28)	0.225
Yes	90 (26.79)	25 (34.72)	
Number of liver metastases			
Single	81 (24.11)	10 (13.89)	0.083
Multiple	255 (75.89)	62 (86.11)	
CA19‐9 level at liver metastasis			
<35 U/mL	168 (50.00)	34 (47.22)	0.766
35~ U/mL	168 (50.00)	38 (52.78)	
CEA level at liver metastasis			
<10 ng/mL	176 (52.38)	32 (44.44)	0.275
10~ ng/mL	160 (47.62)	40 (55.56)	
Liver chemistry score			
Low	166 (49.40)	26 (36.11)	0.055
High	170 (50.60)	46 (63.89)	

*Note*: Data were described as *n* (%) or *median* (*interquartile range*).

Abbreviations: BMI, body mass index; CA19‐9, carbohydrate antigen 19‐9; CEA, carcinoembryonic antigen; ECOG, Eastern Cooperative Oncology Group; OS, overall survival.

### 3.2 Liver chemistry score

**FIGURE 2:**
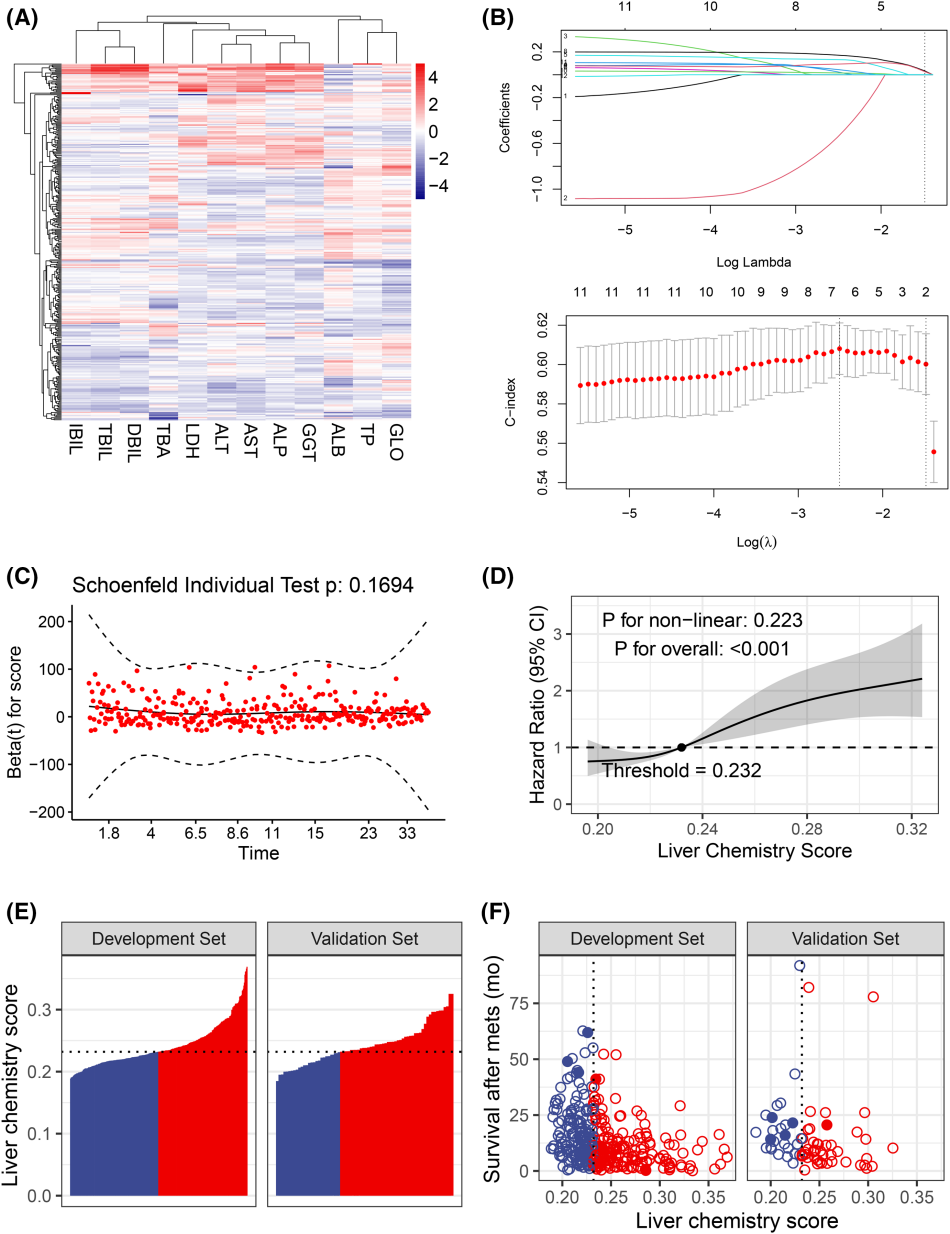
Generation and description of the liver chemistry score. (A) The normalized level of 12 liver chemistry indicators in the development set. All the values were ln‐transformed and normalized. (B) Lasso regression selected AST and ALP based on the penalty parameter that was determined by 10‐fold cross validation following the 1 − SE criterion. (C) The distribution of the liver chemistry score was in accordance with proportional hazard assumption. (D) Restricted cubic spline showed a linear relationship between the liver chemistry score and the survival hazard. (E) The distribution of the liver chemistry score in both cohorts. Blue and red bars indicated low and high score subgroup, respectively. (F) The relationship between the liver chemistry score and the survival in both cohorts. Blue and red dots indicated low and high score subgroups, respectively. Hollow and solid dots indicated dead and alive subjects, respectively. Abbreviations: ALB, albumin; ALP, alkaline phosphatase; ALT, alanine transaminase; AST, aspartate aminotransferase; DBIL, direct bilirubin; GGT, gamma‐glutamyl transpeptidase; GLO, globulin; IBIL, indirect bilirubin; LDH, lactate dehydrogenase; TBA, total bile acid; TBIL, total bilirubin; TP, total protein

The 12 indicators are globally ubiquitous in biochemical tests; therefore, it is clinically feasible to implement a prognostic tool incorporating these markers. Figure 2A illustrated the normalized level of the 12 liver chemistry indicators after ln transformation in the development set. All 12 LFTS were included as predictive variables in the Lasso regression, and survival outcome and survival time were included as dependent variables. The 10‐fold cross validation selected two special lambda values that had similar model performance, to keep the model simplified, we chose the largest lambda with one standard error of C‐index (lambda.1se). In this situation, AST and ALP were selected (Figure 2B). Together with the LASSO regression coefficients (Figure 2B), the LCS was constructed as

$$0.03343515\times \ln\;\left(\mathrm{AST},U/L\right)+0.02687997\times \ln\;\left(\mathrm{ALP},U/L\right).$$

**FIGURE 3:**
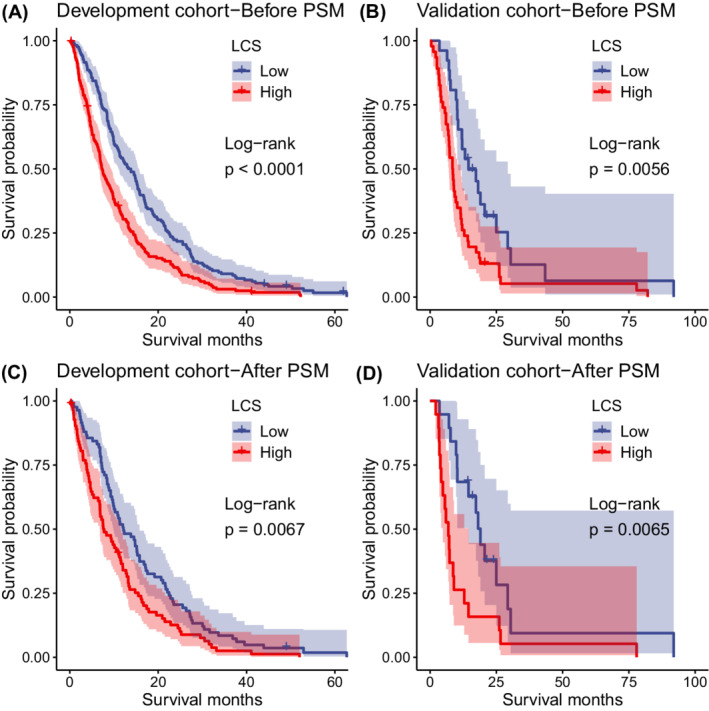
Kaplan–Meier survival curves for low and high liver chemistry score patients in development and validation cohort. (A and B) The Kaplan–Meier survival curve for patients in the low‐score and high‐score groups in the development/validation cohort before PSM. (C and D) The Kaplan–Meier survival curve for patients in the low‐score and high‐score groups in the development/validation cohort after PSM. Shadows indicated 95% CIs for survival probabilities. Abbreviations: LCS, liver chemistry score; PSM, Propensity score matching

The score held PH assumption (Figure 2C) and had a significant impact on survival risk in a linear fashion (Figure 2D). The median and interquartile range of the score in the development and validation set were 0.232 (0.218–0.256) and 0.238 (0.221–0.256), respectively. The RCS indicated that the threshold of the score was 0.232, we chose this point as the cutoff value in the analyses below. Therefore, patients in the two cohorts were further divided into low and high score subgroup. The distribution of the score in both cohorts was shown in Figure 2E. The relationship between the score and survival time was shown in Figure 2F. In Table S1, we analyzed the distribution of clinical pathophysiological parameters in the low‐score and high‐score groups in different cohorts. Considering the differences in the distribution of certain pathophysiological events between patients with high and low scores in the two cohorts, these factors may influence patient survival, we used the PSM method to balance the pathophysiological events between low‐score and high‐score groups in two cohorts. The results after PSM are shown in Table S2.The median OS for patients in low and high score group in the development cohort was 13.54 (95% CI: 11.1–15.6) and 7.3 (6.6–9.3) months, respectively (*p* < 0.001); in the validation cohort, patients with low score also experienced longer survival than patients with high score (mOS: 14.5 [10.6–29.3] vs. 8.5 [7.1–11.5], *p* < 0.0056), and the same result was achieved after PSM (mOS: 12.14 vs. 7.15, *p* < 0.0067 and mOS: 19 vs. 6.9, *p* < 0.0065). Kaplan–Meier survival curves for low and high liver chemistry score patients in difference development and validation cohort is shown in Figure 3.

### 3.3 Cox regression analysis

First, RCS analysis in development dataset resulted “10 ng/mL” and “35 U/mL” as the thresholds for CEA and CA19‐9 level in consideration of their relationship with survival outcome (Figure S1); patients were then divided into two subgroups by above cutoff values. They and other variables were then separately introduced into univariate Cox regression, as shown in Table 2; patients with poor performance status (ECOG 2~), presence of extrahepatic metastasis, multiple liver metastasis, high level of CEA (≥ 10 ng/mL), high level of CA19‐9 (≥35 U/mL), high tumor grade (G3~4) as well as high LCS were at high risk of death while compared to their counters (all *p* values <0.05). These significant variables were included into multivariate Cox analysis; after running forward stepwise procedure, ECOG, extrahepatic metastasis, number of liver metastasis, CA19‐9 level, and LCS were finally retained in the equation (model incl. LCS), although multiple liver metastasis marginally increased death risk (HR = 1.29, 95% CI: 0.99–1.68, *p* = 0.064). As comparison, we also performed a new round of multivariate stepwise Cox regression analysis which excluded LCS (model not incl. LCS); the result was showed in Table S3.

**TABLE 2:** Cox regression analyses in the development cohort (*n* = 336)

Variables	Univariate		Multivariate	
	HR (95% CI)	*p*‐value	HR (95% CI)	*p*‐value
Age at liver metastasis (70~ vs. <70)	0.80 (0.63, 1.01)	0.060	NI	
Sex (male vs. female)	1.19 (0.92, 1.53)	0.192	NI	
BMI at liver metastasis	1.01 (0.99, 1.03)	0.336	NI	
ECOG at liver metastasis (2~ vs. 0~1)	1.69 (1.29, 2.21)	<0.001	1.54 (1.17, 2.02)	0.002
Extrahepatic metastasis (present vs. absent)	1.61 (1.27, 2.04)	<0.001	1.47 (1.16, 1.87)	0.002
Number of liver metastases (multiple vs. single)	1.46 (1.13, 1.89)	0.004	1.29 (0.99, 1.68)	0.064
CEA at liver metastasis (10~ vs. <10 ng/mL)	1.24 (1.00, 1.55)	0.049	NS	
CA19‐9 at liver metastasis (35~ vs. <35 U/mL)	1.45 (1.16, 1.80)	0.001	1.26 (1.00, 1.58)	0.050
Grade (ref: G1‐2)			NS	
G3‐4	1.35 (1.04, 1.76)	0.026		
Unknown	1.24 (0.89, 1.73)	0.201		
Local liver treatment (yes vs. no)	0.75 (0.52, 1.06)	0.106	NI	
Alcohol drinking (yes vs. no/unknown)	0.98 (0.82, 1.17)	0.823	NI	
Chronic liver disease (yes vs. no/unknown)	0.80 (0.41, 1.55)	0.511	NI	
Previous gastrectomy (yes vs. no)	0.89 (0.73, 1.09)	0.257	NI	
Previous hepatotoxic drug exposure (yes vs. no)	1.20 (0.94, 1.53)	0.149	NI	
Liver chemistry score (high vs. low)	1.70 (1.37, 2.12)	<0.001	1.46 (1.16, 1.83)	0.001

Abbreviations: BMI, body mass index; CA19‐9, carbohydrate antigen 19–9; CEA, carcinoembryonic antigen; CI, confidence interval; ECOG, Eastern Cooperative Oncology Group; HR, hazard ratio; NI, not included in analysis; NS, nonsignificant.

### 3.4 Nomogram and validation

**FIGURE 4:**
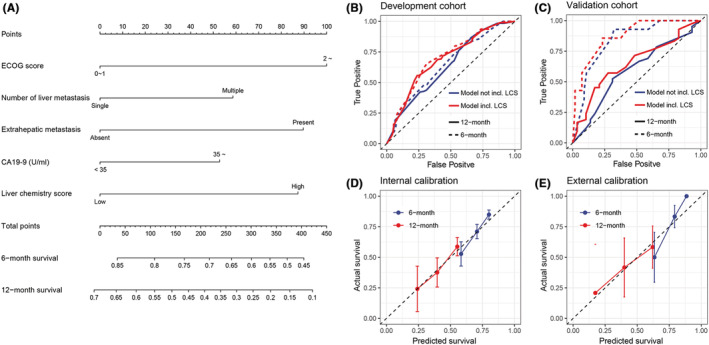
Nomogram including liver chemistry score in predicting survival of gastric cancer after liver metastasis. (A) nomogram incorporated ECOG, number of metastatic lesions in the liver, extrahepatic metastasis, CA19‐9 level, and liver chemistry score to predict 6‐ or 12‐month survival probabilities. (B and C) the 6‐ and 12‐month ROC curves for model incl. and not incl. liver chemistry score in the development and validation cohort. (D and E) the calibration plots in the development and validation cohort

Based on the multivariate Cox regression result, a prognostic nomogram which incorporated ECOG, number of liver metastases, extrahepatic metastasis, CA19‐9 level, and LCS was built, the 6‐ and 12‐month survival rates were predicted as shown in Figure 4A. To validate the performance of the nomogram, time‐dependent ROC curves were depicted; Figures 4B,C showed ROC curves of models for their predicting abilities in development and validation cohort, respectively. While comparing to model not incl. LCS, model incl. LCS showed superior performance. AUC for 6‐month ROC was 0.699 (95% CI: 0.638–0.759) versus 0.647 (0.584~0.711) in development cohort, 0.875 (0.784–0.966) versus 0.837 (0.734–0.940) in validation cohort; the AUC for 12‐month ROC was also higher after introducing LCS to the model (0.687 [0.629–0.745] vs. 0.637 (0.577–0.697) in development cohort and 0.651 [0.523~0.779] vs. 0.591 [0.456~0.725] in validation cohort). In addition, the nomogram predicted survival fit well with actual survival both in the internal and external validation as shown in calibration curves (Figures 4D,E).

## 4 DISCUSSION

In the current study, we generated a prognostic LCS based on profiles of serum LFTs in GCLM patients and validated its ability in distinguishing patients with different prognosis in an independent cohort. By using LASSO regression, two predictive indicators (AST and ALP) were filtered out from a panel of LFTs and total score for each patient was calculated with the equation 0.03343515 × ln (AST, U/L) + 0.02687997 × ln (ALP, U/L). While incorporating the score into prognostic models for GCLM patients, the predictive ability was higher than conventional model (not incl. LCS). This study built a bridge between patients' serum LFTs at liver metastasis and their long‐term survival, the results would be helpful to clinicians for prognosis prediction and decision‐making.

LASSO regression, the short for least absolute shrinkage and selection operator regression, is a method for both feature selection and regularization to improve the accuracy and regularization of statistical models. It is suitable for survival data of high dimension, strong correlation, and small sample.^23^ In the present study, due to the relatively large number of liver chemical indicators and the mutual influence among the indicators, we used the LASSO method to balance the interactions and multicollinearity among the indicators and to prevent over‐fitting. So far as we know, LASSO regression methods for screening clinical biochemical indicators in the study of prognosis of gastric cancer are still rare.

In previous models,^12^, ^13^, ^14^, ^15^, ^16^, ^17^, ^18^, ^19^ parameters of laboratory tests were usually combined with clinical parameters for prognostic predicting, the selection of laboratory parameters into Cox regression equation was arbitrary or only in view of clinical judgment, without consideration of inner connections between variables in one panel of test. In fact, for serum LFTs, the elevation or decrease of liver enzymes is usually resonant especially in cancer patients.^24^ In the development of current model, a panel of 12 biochemical markers in LFTs was introduced into LASSO regression for data dimension reduction, the precipitated LCS can be considered as a surrogate of the profile of panel of LFTs and to be more convenient in clinical application.

The prognostic values of liver enzymes in gastric cancer patients have been explored by previous studies. Elevated ALP,^12^, ^13^, ^14^, ^15^, ^16^, ^17^, ^18^ higher AST/ALT ratio,^25^ decreased albumin,^14^, ^15^, ^16^, ^17^ high serum total bilirubin^14^, ^16^, ^19^ as well as high serum GGT^26^ were shown to be as independent poor prognostic factors of gastric cancer and incorporated into several prognostic models. In consistent with previous studies, we also demonstrated the independent prognostic value of AST and ALP in our cohort. However, most of above studies enrolled patients with advanced gastric cancer with nonspecific organs of metastasis or patients only with specific therapy (e.g., first‐line chemotherapy). Our study focused on subgroup patients with liver metastasis and received palliative chemotherapy. As we know, there is still no prognostic model specifically for this subgroup of patients. More particularly, existing prognostic models mainly developed in Europeans, Koreans, or Japanese, we hereby proposed a model for Chinese GCLM patients, which accounts for the largest proportion of patients in the world.^27^

The C‐indies of nomogram was moderate in this study. We attributed this to the following reasons: (1) the retrospective nature of this study: some of the patient‐related characteristics which may influence expression of liver enzymes, such as fatty liver disease and medication history, was incompletely documented in the patients' files and resulting in bias on the results; (2) other factors that may have impacted on survival of patients were difficult to traceback, such as Her‐2 status of the tumor, latter lines of treatment after liver metastasis, and so on. In addition, this is model employed data from two medical centers; multicentral data are needed to further verify the performance of the model.

## 5 CONCLUSIONS

A liver chemistry score based on AST and ALP was developed and shown ability in separating survival of GCLM patients, nomogram involving poor performance status (ECOG 2~), existence of extrahepatic metastasis, multiple liver metastasis, high level of CA19‐9 (≥ 35 U/mL) and LCS (≥0.232) can be used as tools for individually prognostic predicting.

## AUTHOR CONTRIBUTION

Y.F., C.Z., Z.W., and T.M. designed the study. Y.F., Z.W., M.X., Y.Y., and Y.Z. referred to case files and collected patients' data. H.X., X.Z., and C.F. accomplished follow‐up and vital status determination. Y.F., C.Z., C.F., and J.S. performed the statistical analysis and interpreted the results. C.Z., Y.F., and T.M. prepared the manuscript. All authors reviewed and approved the final manuscript.

## FUNDING INFORMATION

This study was funded by grants from the Anhui Provincial Key Research and Development Program (1804b06020351), the First Affiliated Hospital of Anhui Medical University Clinical Research Project (LCYJ2021YB015), and the Youth Research Foundation of Ma'anshan Municipal People's Hospital (YQ‐2022‐07).

## CONFLICT OF INTEREST

The authors declare that they have no competing interest.

## ETHICAL STATEMENT

All procedures performed in studies involving human participants were in accordance with the 1964 Helsinki Declaration and its later amendments or comparable ethical standards. The protocol was approved by the Ethics Committee of the First Affiliated Hospital of Anhui Medical University (reference number: Quick‐PJ 2021‐05‐19). Due to the retrospective nature of this study, informed consent was waived by the Ethics Committee of the First Affiliated Hospital of Anhui Medical University.

## Supporting information


Figure S1

Table S1

## Data Availability

The datasets generated during the current study are not publicly available due to privacy and ethical restrictions but are available from the corresponding author on reasonable request.
