# Supplementary figures and images for: Incorporation of liver chemistry score in predicting survival of liver‐involved advanced gastric cancer patients who received palliative chemotherapy
Source: Cancer Med. 2022 Sep 4;12(3):2831–41. doi: 10.1002/cam4.5179 (PMC9939141; doi:10.1002/cam4.5179)

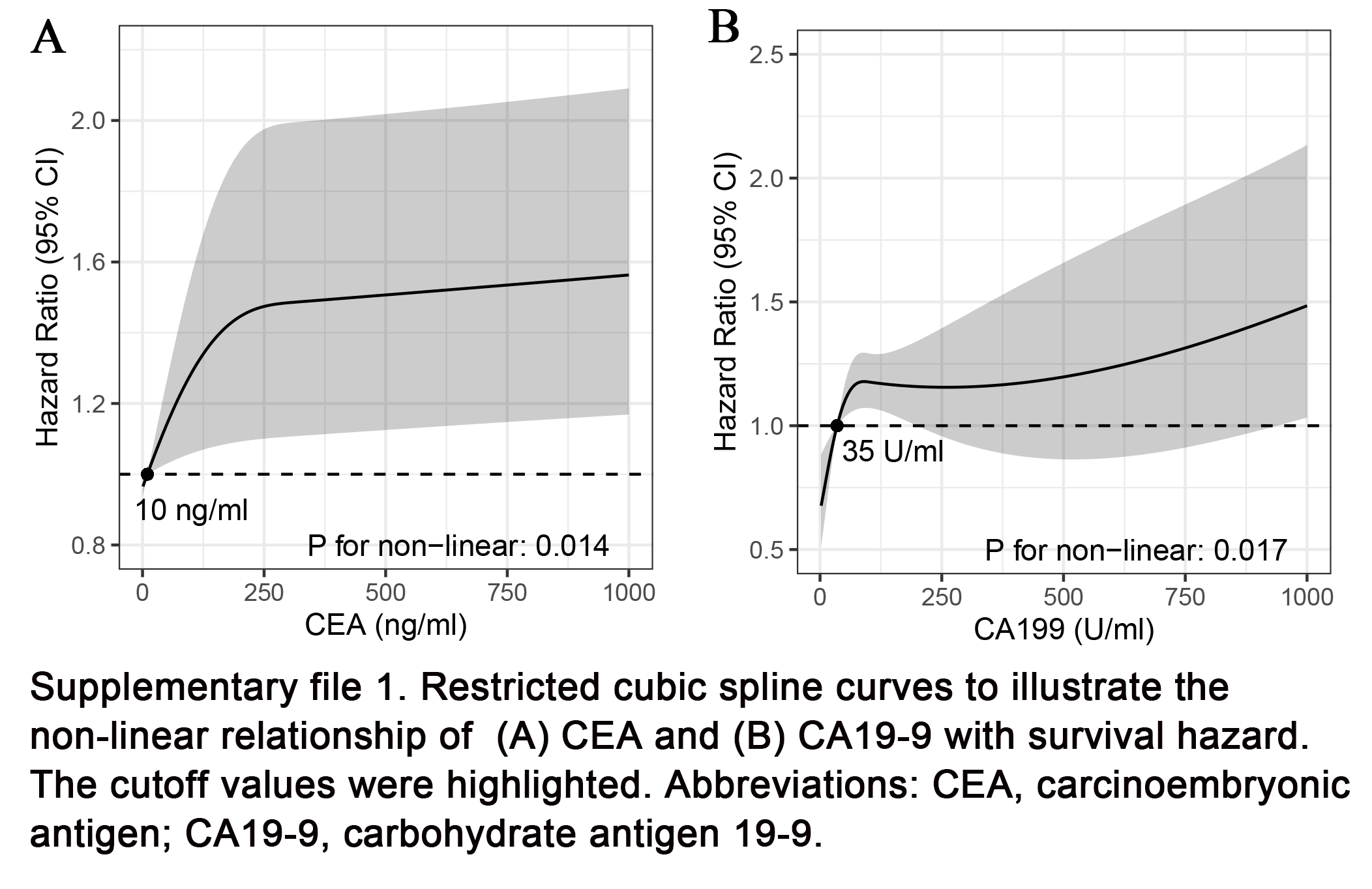

Supplement: Supplementary file 1 — Figure S1 [file CAM4-12-2831-s002.tif]
