# Supplementary material for: Incorporation of liver chemistry score in predicting survival of liver‐involved advanced gastric cancer patients who received palliative chemotherapy
Source: Cancer Med. 2022 Sep 4;12(3):2831–41. doi: 10.1002/cam4.5179 (PMC9939141; doi:10.1002/cam4.5179)
Supplement: Supplementary file 2 — Table S1 [file CAM4-12-2831-s001.docx]

Supplementary table1. Distribution and comparison of clinical characteristics among low and high liver chemistry score groups in the two cohorts.

| Variables | Development cohort (*n*=336) | | *P*-value | Validation cohort (*n*=72) | | *P*-value |
| --- | --- | --- | --- | --- | --- | --- |
|  | Low score (*n*=166) | Hight score (*n*=170) |  | Low score (*n*=26) | Hight score (*n*=46) |  |
| Age at liver metastasis (years) | 64.0 (56.0, 69.8) | 65.5 (60.0, 73.0) | 0.041 | 64.0 (60.5, 70.8) | 67.0 (62.0, 71.0) | 0.546 |
| BMI at liver metastasis (kg/m^2^) | 20.8 (18.6, 22.8) | 20.5 (18.4, 22.2) | 0.605 | 21.2 (19.6, 22.5) | 21.9 (20.1, 24.4) | 0.185 |
| Sex |  |  | 0.123 |  |  | 0.580 |
| Female | 45 (27.1) | 33 (19.4) |  | 4 (15.4) | 11 (23.9) |  |
| Male | 121 (72.9) | 137 (80.6) |  | 22 (84.6) | 35 (76.1) |  |
| Grade |  |  | 0.238 |  |  | **0.034** |
| G1-2 | 43 (25.9) | 38 (22.4) |  | 10 (38.5) | 12 (26.1) |  |
| G3-4 | 96 (57.8) | 92 (54.1) |  | 12 (46.2) | 33 (71.7) |  |
| Unknown | 27 (16.3) | 40 (23.5) |  | 4 (15.4) | 1 (2.2) |  |
| Previous gastrectomy |  |  | **0.022** |  |  | >0.999 |
| No | 74 (44.6) | 98 (57.6) |  | 16 (61.5) | 27 (58.7) |  |
| Yes | 92 (55.4) | 72 (42.4) |  | 10 (38.5) | 19 (41.3) |  |
| ECOG score at liver metastasis |  |  | 0.569 |  |  | 0.999 |
| 0~1 | 135 (81.3) | 133 (78.2) |  | 20 (76.9) | 35 (76.1) |  |
| 2~ | 31 (18.7) | 37 (21.8) |  | 6 (23.1) | 11 (23.9) |  |
| Extrahepatic metastasis |  |  | 0.116 |  |  | 0.868 |
| Absent | 57 (34.3) | 44 (25.9) |  | 10 (38.5) | 20 (43.5) |  |
| Present | 109 (65.7) | 126 (74.1) |  | 16 (61.5) | 26 (56.5) |  |
| Local treatment for hepatic lesion |  |  | 0.063 |  |  | 0.526 |
| No | 143 (86.1) | 158 (92.9) |  | 18 (69.2) | 27 (58.7) |  |
| Yes | 23 (13.9) | 12 (7.1) |  | 8 (30.8) | 19 (41.3) |  |
| History of alcohol drinking |  |  | 0.923 |  |  | 0.641 |
| No/unknown | 138 (83.1) | 143 (84.1) |  | 13 (50.0) | 27 (58.7) |  |
| Yes | 28 (16.9) | 27 (15.9) |  | 13 (50.0) | 19 (41.3) |  |
| Chronic liver disease |  |  | 0.999 |  |  | 0.549 |
| No/unknown | 162 (97.6) | 165 (97.1) |  | 26 (100) | 43(93.5) |  |
| Yes | 4 (2.4) | 5 (2.9) |  | 0 (0.0) | 3 (6.5) |  |
| Previous cytotoxic drugs exposure |  |  | 0.465 |  |  | 0.786 |
| No | 125 (75.3) | 121 (71.2) |  | 18(69.2) | 29(63) |  |
| Yes | 41 (24.7) | 49 (28.8) |  | 8 (30.8) | 17 (37.0) |  |
| Number of liver metastases |  |  | **<0.001** |  |  | 0.528 |
| Single | 57 (34.3) | 24 (14.1) |  | 5(19.2) | 5(10.9) |  |
| Multiple | 109 (65.7) | 146 (85.9) |  | 21 (80.8) | 41 (89.1) |  |
| CA19-9 level at liver metastasis |  |  | **0.006** |  |  | 0.113 |
| < 35 U/ml | 96 (57.8) | 72 (42.4) |  | 16(61.5) | 18(39.1) |  |
| 35 ~ U/ml | 70 (42.2) | 98 (57.6) |  | 10 (38.5) | 28 (60.9) |  |
| CEA level at liver Metastasis |  |  | **0.001** |  |  | 0.146 |
| < 10 ng/ml | 102 (61.4) | 74 (43.5) |  | 15 (57.7) | 17(37) |  |
| 10 ng/ml ~ | 64 (38.6) | 96 (56.5) |  | 11 (42.3) | 29 (63.0) |  |

Abbreviations: BMI, body mass index; OS, overall survival; ECOG, Eastern Cooperative Oncology Group; CEA, carcinoembryonic antigen; CA19-9, carbohydrate antigen 19-9.

Supplementary table2. Distribution of clinical characteristics in the development and validation cohort after propensity score matching

| Variables | Development cohort (*n*=166) | |  | Validation cohort (*n*=38) | | *P*-value |
| --- | --- | --- | --- | --- | --- | --- |
|  | Low score (*n*=83) | Hight score (*n*=83) | *P*-value | Low score (*n*=19) | Hight score (*n*=19) |  |
| Age at liver metastasis (years) | 64.0 (58.0, 69.0) | 65.0 (58.0, 70.5) | 0.760 | 63.0 (61.0, 69.5) | 67.00 (63.0, 73.5) | 0.334 |
| BMI at liver metastasis (kg/m^2^) | 20.9 (18.5, 22.9) | 19.83 (17.9, 21.8) | 0.083 | 21.3 (19.9, 22.5) | 21.6 (20.1, 23.3) | 0.827 |
| Sex |  |  | 0.456 |  |  | 0.094 |
| Female | 21 (25.3) | 16 (19.3) |  | 1 (5.3) | 6 (31.6) |  |
| Male | 62 (74.7) | 67 (80.7) |  | 18 (94.7) | 13 (68.4) |  |
| Grade |  |  | 0.860 |  |  | 1.000 |
| G1-2 | 26 (31.3) | 23 (27.7) |  | 7 (36.8) | 6 (31.6) |  |
| G3-4 | 39 (47.0) | 40 (48.2) |  | 12 (63.2) | 13 (68.4) |  |
| Unknown | 18 (21.7) | 20 (24.1) |  |  |  |  |
| Previous gastrectomy |  |  | 1.000 |  |  | 0.737 |
| No | 45 (54.2) | 45 (54.2) |  | 11 (57.9) | 13 (68.4) |  |
| Yes | 38 (45.8) | 38 (45.8) |  | 8 (42.1) | 6 (31.6) |  |
| ECOG score at liver metastasis |  |  | 1.000 |  |  | 1.000 |
| 0~1 | 67 (80.7) | 66 (79.5) |  | 14 (73.7) | 13 (68.4) |  |
| 2~ | 16 (19.3) | 17 (20.5) |  | 5 (26.3) | 6 (31.6) |  |
| Extrahepatic metastasis |  |  | 0.592 |  |  | 0.737 |
| Absent | 23 (27.7) | 19 (22.9) |  | 8 (42.1) | 6 (31.6) |  |
| Present | 60 (72.3) | 64 (77.1) |  | 11 (57.9) | 13 (68.4) |  |
| Local treatment for hepatic lesion |  |  | 0.430 |  |  | 0.737 |
| No |  |  |  | 13 (68.4) | 11 (57.9) |  |
| Yes | 10 (12.0) | 6 (7.2) |  | 6 (31.6) | 8 (42.1) |  |
| History of alcohol drinking |  |  | 0.840 |  |  | 0.192 |
| No/unknown | 67 (80.7) | 69 (83.1) |  | 8 (42.1) | 13 (68.4) |  |
| Yes | 16 (19.3) | 14 (16.9) |  | 11 (57.9) | 6 (31.6) |  |
| Chronic liver disease |  |  | 1.000 |  |  | 1.000 |
| No/unknown | 80 (96.4) | 81 (97.6) |  | 19 (100) | 18 (94.7) |  |
| Yes | 3 (3.6) | 2 (2.4) |  | 0 (0.0) | 1 (5.3) |  |
| Previous cytotoxic drugs exposure |  |  | 0.734 |  |  | 1.000 |
| No | 60 (72.3) | 57 (68.7) |  | 12 (63.2) | 13 (68.4) |  |
| Yes | 23 (27.7) | 26 (31.3) |  | 7 (36.8) | 6 (31.6) |  |
| Number of liver metastases |  |  | 0.825 |  |  | 0.656 |
| Single | 13 (15.7) | 11 (13.3) |  | 4 (21.1) | 2 (10.5) |  |
| Multiple | 70 (84.3) | 72 (86.7) |  | 15 (78.9) | 17 (89.5) |  |
| CA19-9 level at liver metastasis |  |  | 0.535 |  |  | 0.194 |
| < 35 U/ml | 38 (45.8) | 43 (51.8) |  | 12 (63.2) | 7 (36.8) |  |
| 35 ~ U/ml | 45 (54.2) | 40 (48.2) |  | 7 (36.8) | 12 (63.2) |  |
| CEA level at liver Metastasis |  |  | 0.535 |  |  | 0.194 |
| < 10 ng/ml | 40 (48.2) | 45 (54.2) |  | 12 (63.2) | 7 (36.8) |  |
| 10 ng/ml ~ | 43 (51.8) | 38 (45.8) |  | 7 (36.8) | 12 (63.2) |  |

Abbreviations: BMI, body mass index; OS, overall survival; ECOG, Eastern Cooperative Oncology Group; CEA, carcinoembryonic antigen; CA19-9, carbohydrate antigen 19-9.

Supplementary table3. Multiple Cox regression analysis in the development cohort that excluded the liver chemistry score (*n* = 336).

| Variable | *HR (95% CI)* | *P*-value |
| --- | --- | --- |
| ECOG at liver metastasis (2~ vs. 0~1) | 1.57 (1.20, 2.06) | 0.001 |
| Extrahepatic metastasis (yes vs. no) | 1.52 (1.20, 1.94) | <0.001 |
| Number of liver metastases (multiple vs. single) | 1.40 (1.08, 1.82) | 0.011 |
| CA19-9 at liver metastasis (35~ vs. <35 U/ml) | 1.33 (1.06, 1.67) | 0.012 |

Abbreviations: HR, hazard ratio; CI, confidence interval; ECOG, Eastern Cooperative Oncology Group; CA19-9, carbohydrate antigen 19-9.
